# REGγ ablation impedes dedifferentiation of anaplastic thyroid carcinoma and accentuates radio-therapeutic response by regulating the Smad7-TGF-β pathway

**DOI:** 10.1038/s41418-019-0367-9

**Published:** 2019-06-26

**Authors:** Chan Jiao, Lin Li, Pei Zhang, Li Zhang, Ke Li, Riqun Fang, Lei Yuan, Kaixuan Shi, Linan Pan, Qiannan Guo, Xiao Gao, Geng Chen, Shichen Xu, Qingwei Wang, Di Zuo, Wei Wu, Shanlou Qiao, Xiaoshuang Wang, Robb Moses, Jianru Xiao, Lei Li, Yongyan Dang, Xiaotao Li

**Affiliations:** 1 Shanghai Key Laboratory of Regulatory Biology, Institute of Biomedical Sciences, School of Life Sciences, East China Normal University, 500 Dongchuan Road, Shanghai, 200241 China; 2 Department of Orthopedic Oncology, Changzheng Hospital, The Second Military Medical University, 415 Fengyang Road, Shanghai, 200003 China; 3 Department of Pathology, the Second Chengdu Municipal Hospital, Chengdu, 610017 China; 4 Jiangsu Institute of Nuclear Medicine, Key laboratory of Nuclear Medicine, Ministry of Health, 20 Qian Rong Road, Wuxi, Jiangsu 214063 China; 5 Institute of Life & Health Sciences, Chubu University, 1200 Matsumoto-cho, Kasugai, Aichi 487-8501 Japan; 6 Department of Molecular and Cellular Biology, Dan L. Duncan Cancer Center, Baylor College of Medicine, One Baylor Plaza, Houston, TX 77030 USA

**Keywords:** Oncogenes, Endocrine system and metabolic diseases

## Abstract

Anaplastic thyroid cancer (ATC) is the most aggressive human thyroid malignancy, characterized by dedifferentiation and resistance to radioiodine therapy. The underlying mechanisms regulating ATC dedifferentiation are largely unknown. Here, we show that REGγ, a noncanonical proteasome activator highly expressed in ATC, is an important regulator of differentiation in ATC cells. Ablation of REGγ significantly restored expression of thyroid-specific genes, enhanced iodine uptake, and improved the efficacy of ^131^I therapy in ATC xenograft models. Mechanistically, REGγ directly binds to the TGF-β signaling antagonist Smad7 and promotes its degradation, leading to the activation of the TGF-β signal pathway. With gain- and loss-of-function studies, we demonstrate that Smad7 is an important mediator for the REGγ function in ATC cell dedifferentiation, which is supported by expression profiles in human ATC tissues. It seems that REGγ impinges on repression of thyroid-specific genes and promotion of tumor malignancy in ATC cells by activating the TGF-β signal pathway via degradation of Smad7. Thus, REGγ may serve as a novel therapeutic target for allowing radioiodine therapy in anaplastic thyroid cancer patients with poor prognosis.

## Introduction

Anaplastic thyroid cancer (ATC) is one of the most deadly human diseases. Due to its invasive nature and resistance to radiotherapy, ATC has an extremely low cure rate with a median survival of less than 6 months [1], responsible for more than half of all thyroid cancer deaths [2]. However, patients with differentiated thyroid carcinoma have an excellent 10-year survival ranging between 80 and 95% [3]. Radioactive iodine therapy improves the survival rate of patients with differentiated metastastatic thyroid cancer. The sodium iodide symporter (NIS), a transmembrane glycoprotein in the thyroid follicular cells, catalyzes the accumulation of iodide in the thyroid gland, allowing the treatment of differentiated thyroid cancers by radioactive iodine [4]. However, ATC fails to express adequate amounts of thyroid-specific genes, primarily due to *NIS* [5], leading to its resistance to radioiodine therapy. Therefore, innovative approaches for recovery of NIS expression in poorly differentiated thyroid cancers might promote therapy via iodine uptake [6].

TGF-β functions as a tumor promoter through increasing tumor cell invasion and metastasis in late-stage cancers. TGF-β1 is overexpressed in ATC and silencing TGF-β1 inhibits cell migration and invasion of ATC cells [7]. Smad3 activation inhibits expression of Pax8 and its DNA-binding activity, mediating TGF-β-induced downregulation of NIS in thyroid follicular cells [8]. BRAF appears to induce secretion of TGF-β in human PTC and inhibit *NIS* expression [9], substantiating that TGF-β plays an important role in thyroid cancer progression.

REGγ (also known as PA28γ, PSME3, Ki antigen) belongs to the 11 S family of proteasome activators to promote ubiquitin and ATP-independent degradation of proteins [10, 11]. REGγ regulates cell cycle, inflammation, angiogenesis, and additional biological processes [12–16]. In addition, REGγ is overexpressed in several tumors, including thyroid cancer, displaying oncogenic actions [17–19]. However, it is unclear if overexpressed REGγ in ATC promotes its malignancy.

In this study, we demonstrate that REGγ enhances dedifferentiation of ATC cells. Depletion of REGγ restored the expression of thyroid-specific genes in ATC cells and improved radioiodine uptake in vitro and in vivo, therefore, improving ^131^I therapy in ATC xenograft tumors. REGγ mediates upregulation of the TGF-β pathway by degrading Smad7, since inactivation of Smad7 prevents the recovery of thyroid-specific genes in REGγ-deficient ATC cells. Thus, inhibition of the REGγ proteasome might be a promising approach for ATC patients.

## Methods

### Cells

K18 ATC and HEK293T cells were purchased from American Type Culture Collection (ATCC, USA). SW1736 ATC was from James A. Fagin’s laboratory. The REGγ knockdown stable cell lines were generated by integration of retroviral ShRNA vector specific for REGγ to produce ShR (ShRNA against REGγ) or a negative control from OriGene (Rockville, MD) to produce ShN (ShRNA as a negative control) cells. ATC cell lines and HEK293T cell line were cultured in the 1640 and DMEM medium supplied with 10% fetal bovine serum (Gibco), respectively. The 293-REGγ inducible cell lines were previously generated.

### Plasmids, constructs, and expression

HA-REGγ (pcDNA3.1), Flag-Smad7 (pcDNA3.1), PSG5-HA-Smad7, plvx-GFP-Smad7, plvx-Luc-G418, and NIS promoter luciferase (NIS-Luc) reporter plasmid (pGL2-Basic) containing the −2000/ + 375 sequence of NIS promoter were constructed in our laboratory. Smad3 siRNA (F-5′-CCAGUGACCACCAGAUGAA-3′) and Smad7 siRNA (F-5′-CUCUCUGGAUAUCUUCUAUTT-3′ and R-5′-AUAGAAGAUAUCCAGAGAGTT-3′) were synthesized by Genepharma. Plasmids or siRNA were transfected to different cells and cultured for 36 h or 72 h.

### In vitro ^131^I uptake of ATC cells

Overall, 5 × 10^5^ ShN and ShR ATC cancer cells (SW1736 and K18) were plated in triplicates in 12-well plates. After washing with cold HBSS three times, cells were incubated for the indicated time at 37 °C with 1 ml of HBSS containing 1 μCi carrier-free Na^131^I and 10 μM NaI. In control groups, cells were treated with 300 μM NaClO_4_, a competitive inhibitor of NIS, for 30 min to determine the nonspecific radioiodine uptake. Then, cells were washed with ice-cold HBSS for three times, lysed in 1 ml 0.33 M NaOH. The radioactivity was measured with a Perkinelmer 2470 gamma-counter.

### Luciferase assays

SW1736 and K18 ATC cells were washed with cold PBS three times after transfection with NIS-Luc reporter for 36 h, harvested in the lysis buffer provided in the Luciferase Assay Kit (Promega). After one cycle of freezing and thawing, the cell lysates were centrifuged at 12,000 rpm for 10 min at 4 °C. Then 20 μl of supernatant was added to an equal amount of luciferase assay substrate. Luminescence was measured as relative light units using the LUMIstar OPTIMA (BMG Labtech) illuminometer.

### Western blot analysis, immunoprecipitation, and in vitro proteolytic analysis

Cells were collected in NP40 lysis buffer and minced tissues were lysed in RIPA buffer on ice for 15 min. For NP40 lysed samples, protein concentrations were determined by BCA assay kit (Beyotime, China). Equal amount of proteins were run on a 10–12% SDS–PAGE, transferred to a nitrocellulose membrane (Millipore, MA, USA), and then immunoblotted with the NIS (Millipore and Proteintech 24324-1-AP), Pax8 (Millipore and Bioworld BS3459), REGγ, p-Smad3, Smad3 (Proteintech), Smad7 (Abcam ab55493 and Proteintech), or β-actin antibodies (CST 3102 and Sigma A5441) overnight. After incubation with secondary fluorescent antibodies for 1 h, the antibody-bound proteins were analyzed by a fluorescent western blot imaging system (Odyssey).

For co-immunoprecipitation assay, HEK293 cells were transiently transfected with plasmids expressing pcDNA3.1-HA-REGγ and pcDNA3.1-Flag-Smad7 for 48 h. Then, cells were harvested in CHAPS lysis buffer. Extracts were incubated overnight with 2.5 µg of anti-HA or anti-Flag (Santa Cruz Biotechnology, Heidelberg, Germany) antibody in the presence of Protein G beads from a Protein G Immunoprecipitation kit (Sigma-Aldrich, Buchs, Switzerland). Complexes were washed, denatured, and eluted for western blot analysis.

For in vitro proteolytic analysis, Smad7 protein was generated by in vitro translation [10]. The protein degradation experiment was conducted by incubating Smad7, 20S proteasome (Boston Biochem), and purified REGγ for 3–6 h in 25-μl reaction volume at 30 °C with appropriate controls. The results were analyzed by western blotting.

### Quantitative real-time PCR

The total RNA was extracted from cells or tumor tissues using TRIZOL reagent (TaKaRa). A pool of cDNA was synthesized from 1 μg of RNA with M-MLV reverse transcriptase (Takara Co., Otsu, Japan) as described [17]. Real-time PCR was performed using SYBR Premix Ex Taq (TaKaRa). The following parameters were used for the PCR: 95 °C for 10 min followed by 40 cycles at 95 °C for 30 s, 55 °C for 30 s, and 72 °C for 45 s. Gene expression was normalized against 18S RNA. The primers used in this study are listed in Supplementary Table 1.

### Immunofluorescence analysis

SW1736 and K18 ATC cancer cells were fixed with 4% paraformaldehyde for 15 min and permeabilized with 0.01% Triton X-100 for 15 min. After blocking with 1% bovine serum in PBS for 30 min, cells were incubated with one of the following antibodies: NIS, Pax8, Smad7, Smad3, and Smad4 (Abcam) diluted in PBS overnight at 4 °C. Then, cells were washed with PBS three times followed by incubation with 1:500 Alexa Fluor 550 and Alexa Fluor 488 phalloidin-conjugated secondary antibodies (Invitrogen) for 1 h. Cells were stained with DAPI for 1 min. Finally, cells were mounted on glass slides in the AquaPoly/Mount medium (Polysciences). Photomicrographs were recorded by an Olympus microscope. The percentage of positive cells was determined after counting at least 300 cells in a double-blinded manner.

### HE staining and immunohistochemical analysis

Human ATC samples were fixed in 4% paraformaldehyde, dehydrated with ethanol, and then fixed in paraffin. Approximately 4-μm-thick sections were cut and deparaffinized, rehydrated, and stained with hematoxylin–eosin (H&E). Stained slides were then evaluated using IX81 microscopy (Olympus, Tokyo, Japan).

Immunohistochemical analysis was performed with streptavidin–biotin complex (ABC) approach following the instruction by a Neobioscience kit (Shenzhen, China). Primary antibodies were diluted in PBS as follows: anti-REGγ, anti-NIS, anti-SMAD7, and anti-Pax8. Prior to antibody incubation, an antigen was retrieved in 10 mM sodium citrate, pH 6.0, for 15 min at 100 °C in a water bath. Color reaction was visualized after addition of diaminobenzidine (DAB)-H_2_O_2_ as a substrate for peroxidase. All sections were counterstained with hematoxylin, dehydrated, mounted, and observed under a microscope. Staining percentage was classified as negative (0–25%), weak (25–50%), moderate (50–75%), or strong (75–100%) according to previous publication [20].

### Xenograft mouse models

ATC cells (5 × 10^6^ in 0.1 mL of PBS per mouse) were inoculated subcutaneously on the right back sides of the mice. In all, 100 μl of luciferase substrate at the concentration of 33 mg/ml was injected into the intraperitoneal cavity, ~5 min before imaging. Mice were anesthetized with isoflurane and then ventral images were collected for 30 s to 2 min using the IVIS (Xenogen Corp., Alameda, CA). Photons emitted from the primary tumor and lung region were quantified using Living Image software (Xenogen Corp., Alameda, CA).

### ^131^I uptake and therapy of xenograft tumors in nude mice

Two-month-old BALB/c nude mice were used in xenograft and radiotherapy studies following the ethical and safety guideline approved by the Animal Center at Jiangsu Institute of Nuclear Medicine.

SW1736 and K18 (ShN and ShR) ATC cells with a stably integrated luciferase reporter gene (effluc) were subcutaneously implanted into the dorsal sides (ShN to left and ShR to the right side) of BALB/c nude mice in two groups (six mice in each group). For transplanted tumors sized 6–8 mm in diameter, Cherenkov imaging was performed for 30 min after Na^131^I (0.5 mCi) i.p. injection.

For ^131^I therapy, mice were administered 1.5 mCi Na^131^I by a single i.p. injection. The control mice were administered 0.9% NaI. Tumors were measured before administration of radioiodine and weekly thereafter. Tumor optical images were acquired by the IVIS imaging system, and tumor size was quantified with Living Image software.

### Bioinformatics analysis

The samples of ATC (*n* = 20) and PTC (*n* = 17) were chosen from the NCBI Gene Expression Omnibus (http://www.ncbi.nlm.nih.gov/geo) to determine the transcript levels of *REGγ, NIS, Pax8*, and *Smad7*, the accession number is GSE76039.

### ATC patient samples

Ten unnamed human ATC samples were collected from the Second Chengdu Municipal Hospital in China. Patient organization and case access are in agreement with the ethical guidelines and requirements by the hospital.

### Statistical analysis

All experiments were performed independently at least three times. All data were expressed as the mean ± SD. Paired Student's *t* test or one-way ANOVA were used to compare the differences in the data between two groups or more. *P*-value of less than 0.05 were considered to be of significance.

## Results

### REGγ deficiency augments ^131^I uptake in ATC cells

**Fig. 1:**
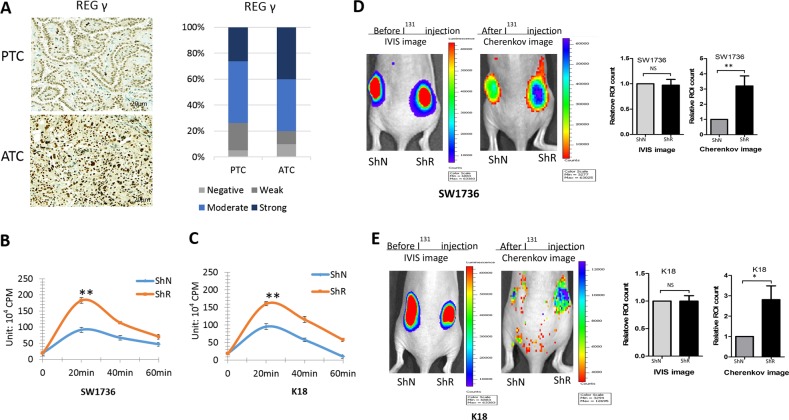
REGγ deficiency augments radioiodine uptake in human ATC cells. **a** REGγ expression was markedly higher in human ATC tissues than PTC tissues. IHC immunostaining (left) and quantitative analyses (right) of REGγ expression were performed in 10 ATC and 19 PTC patients. Scale bar, 20 μm. **b**, **c** Time course of ^131^I uptake in ShN and ShR SW1736 (**b**) and K18 (**c**) cells. Values represented the results of three independent experiments, and were expressed as mean ± SEM. **p < 0.01, ShR vs. ShN. **d**, **e** Xenograft tumors generated from ShR cells had expedited absorption of radioiodine than tumors from ShN cells. Images of tumors from ShR and ShN SW1736-luc (d, *n* = 28) or K18-luc cells (e, *n* = 24) on the left represent bioluminescence of luciferase activities, reflecting the tumor size of implantation of tumors upon injection. The images of iodine uptake on the right panels were taken by a cherenkov camera 30 min after ^131^I intraperitoneal injection. Luminescence signal intensity was quantified by a region of interest (ROI) analysis and expressed as photons per second per cm^2^ from mice. The statistical analysis for IVIS ROI values (left) and cherenkov ROI vuales (right) were presented in graphical form. The data were represented as the mean ± SEM. *p < 0.05, **p < 0.01, ShR vs. ShN

Previous studies [21] reported anomalous REGγ expression in thyroid neoplasms, with higher levels in poorly differentiated than in well-differentiated thyroid cancers. To investigate REGγ function in ATC, we analyzed histobiochemistry in ten samples by comparison with papillary thyroid cancer (PTC). REGγ displayed nuclear localization in the ATC and PTC tissues. Critically, REGγ expression in ATC was found higher than in PTC (Fig. 1a), suggesting that REGγ may play a role in the development of poorly differentiated ATC. Therefore, we addressed whether hyperactivation of REGγ might be involved in the dedifferentiation of ATC.

First, we determined if attenuation of REGγ in ATC cells may restore radioiodine uptake. Approximately 20 min after ^131^I treatment, REGγ-depleted SW1736-ShR and K18-ShR cells had 94.7% and 84.2% higher radioiodine absorption, respectively, than the ShN control cells (Fig. 1b, c). Then, to substantiate the influence of REGγ on iodine intake, we generated xenograft tumors with similar volume and monitored radioiodine absorption in vivo (Fig. 1d, e). Compared with the ShN controls, REGγ knockdown greatly enhanced the uptake of iodine in the xenograft tumors, with an increase by 3.2- and 2.8-fold for SW1736-ShR and K18-ShR tumors, respectively (Fig. 1d, e). The results suggest the possibility that REGγ may be involved in dedifferentiation of ATC cells.

### Ablation of REGγ restores expression of thyroid-specific genes in ATC cells

**Fig. 2:**
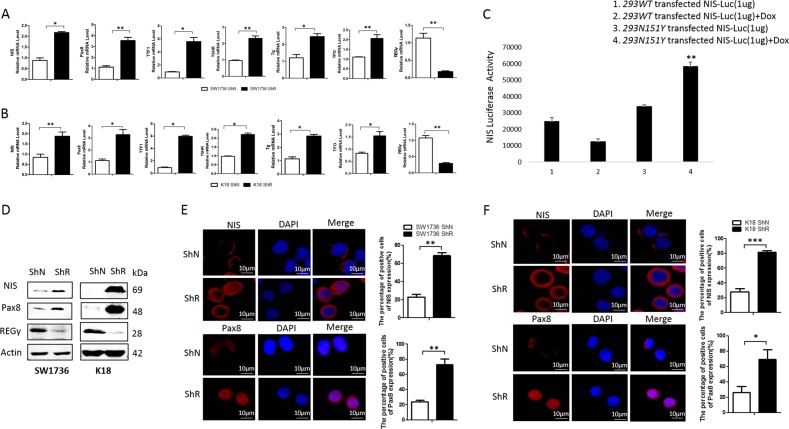
Abrogation of REGγ restores expression of thyroid-specific genes in ATC cells. **a** Cells with REGγ stable knockdown (ShR) had increased expression of *NIS*, *Pax8*, *TSHR*, *TTF1, Tg*, and *TPO* when compared with ShN cells. Real-time PCR analyses were performed in SW1736 ShR and control (ShN) ATC cells and expressed as the relative mRNA levles between ShR and ShN cells. **b** Expression of thyroid-specific genes was analyzed by real-time PCR in ShN and ShR K18 ATC cells. **c** Inactivation of REGγ elevates *NIS* transcription. NIS-Luc reporter assays were performed in HEK293 cells inducibly expressing wild-type (WT) or a dominant-negative mutant REGγ in the presence or absence of 1 μg/ml doxycycline for 36 h. **d** Silencing REGγ increased NIS and Pax8 protein levels in SW1736 and K18 ShR cells. **e**, **f** Immunofluorescence analysis of Pax8 and NIS in SW1736 (**e**) and K18 (**f**) ATC cells before and after REGγ depletion. The right panels were the quantitative results of Pax8- and NIS-positive cells. A minimum of 300 cells per sample were counted in triplicate. The data were represented as the mean ± SEM of three independent experiments. *p < 0.05, **p < 0.01, ***p < 0.001, ShR vs. ShN

To further determine if REGγ regulates dedifferentiation of ATC cells, we evaluated expression of thyroid-specific genes following RNAi of REGγ. In contrast to the control in ShN cells, expression of *TTF1*, *Pax8*, *NIS*, *Tg, TSHR*, and *TPO* in SW1736-ShR and K18-ShR was markedly upregulated (Fig. 2a, b). The effects of REGγ on *NIS* transcription were also investigated using a *NIS* luciferase (luc) reporter construct in a Doxycycline-inducible cellular system to overexpress either a wild-type (WT) REGγ or a dominant-negative loss-of-function mutant REGγ (N151Y). Overexpression of REGγ greatly inhibited *NIS*-luc reporter activity, while the N151Y-mutant failed to do so (Fig. 2c; Supplementary Fig. 1A).

Western blot analysis validated the increase in NIS and Pax8 protein expression in SW1736 and K18 ATC cells, with stable knockdown of REGγ (Fig. 2d). Furthermore, immunofluorescent analyses displayed more intensive staining of NIS and Pax8 in ATC-ShR cells than in ATC-ShN cells (Fig. 2e, f). Together, our results indicate that blockade of REGγ action has the potential to induce re-differentiation of ATC cells, as demonstrated by restoration of NIS transcription.

### REGγ suppresses thyroid-specific genes by promoting the TGF-β/Smad signal pathway

**Fig. 3:**
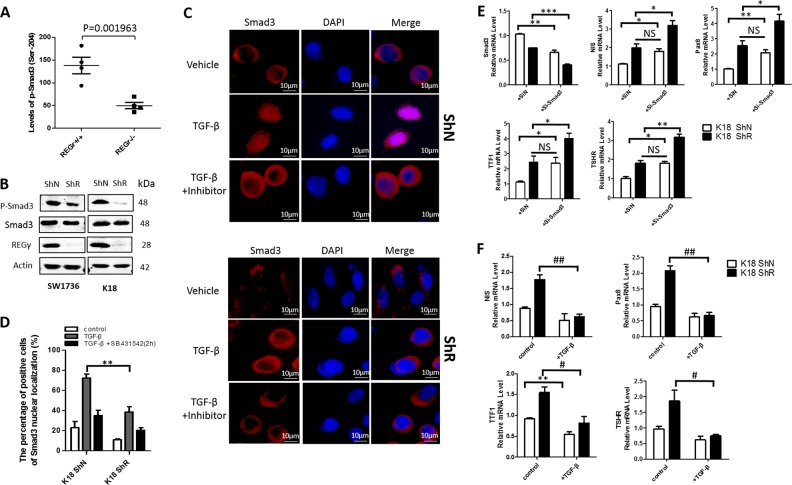
REGγ suppresses thyroid-specific genes by promoting the TGF-β/Smad signal pathway. **a** Antibody array analysis disclosed about threefold higher levels of p-Smad3 (ser204) in REGγ WT cells than in REGγ-deficient MEF cells. **b** The amount of p-Smad3 protein expression was compared between ShN and ShR SW1736 and K18 ATC cells after 5 ng/ml TGF-β for 2 h by western blotting analysis. **c** Immunofluorescent staining of Smad3 in K18 ShN and ShR cells before (serum starved for 24 h) and after TGF-β (5 ng/ml for 6 h) in the presence or absence of 10 μM TGF-β receptor inhibitor SB431542. Nuclei were stained with DAPI. Scale bar, 10 μm. **d** The quantitative results for Fig. 3c. The data were represented as the mean ± SEM of three independent experiments. **p < 0.01, ShR vs. ShN. **e** Silencing Smad3 in K18 ShN ATC cells increased the expression of *NIS*, *Pax8*, *TSHR*, and *TTF1* to levels equivalent to the untreated ShR cells. Cells were transfected with siRNA against Smad3 for 48 h, and the expression of *NIS*, *Pax8*, *TSHR*, and *TTF1* were detected with real-time PCR. The data were represented as the mean ± SEM of three independent experiments. *p < 0.05, **p < 0.01, ***p < 0.001, siSmad3 vs. control. **f** TGF-β treatments inhibited thyroid-specific gene expression in K18 ShN and ShR ATC cells. Cells were serum starved for 24 h, then treated with or without 5 ng/ml TGF-β for 6 h. The mRNA expression of *NIS*, *Pax8*, *TSHR*, and *TTF1* was detected by real-time PCR. The data were represented as the mean ± SEM of three independent experiments. *N* = 3, *p* < 0.05. * = TGF-β compared with ShN Control, ^#^ = TGF-β compared with ShR Control

To address the molecular mechanism by which REGγ negatively regulates thyroid-specific genes, we performed a high-throughput proteomic screen of REGγ targets or effectors by antibody array analysis (FullMoon BioSystem) [15]. Among the top ten proteins differentially expressed in REGγ WT and REGγ KO MEFs, we found approximately threefold higher levels of active Smad3 (phosphor-Ser 204) [22] in WT than in KO cells (Fig. 3a). We performed western blot analysis to confirm the correlation between REGγ and p-Smad3 in ATC cells. Consistently, the p-Smad3 levels were markedly higher in ShN SW1736 and K18 ATC cells than in ShR cells (Fig. 3b). Moreover, we validated that nuclear Smad3 is much more abundant in REGγ containing ShN than in REGγ-silenced ShR ATC cells following TGF-β treatments (Fig. 3c, d; Supplementary Fig. 1B–C). In contrast, SB-431542, an inhibitor of the TGF-β type I receptor, abolished nuclear accumulation of Smad3 in all the cells (Fig. 3c, d; Supplementary Fig. 1B–C), reminiscent of REGγ ablation. Silencing Smad3 markedly increased the expression of *NIS*, *Pax*8, *TTF1*, and *TSHR* in K18 and SW1736 ATC cells (Fig. 3e; Supplementary Fig. 1D), consistent with a previous report that TGF-β/Smad3 signaling is a potent repressor of *Pax8*/*NIS* in normal epithelial thyroid cells [8]. To substantiate the role of Smads on NIS expression, *NIS* luciferase reporter assays were performed in the presence of Smad2 or Smad3. Either greatly inhibited the activitiy of the *NIS* reporter in SW1736 or K18 ATC cells (Supplementary Fig. 1E).

Indeed, TGF-β signal transduction is crippled in ShR cells since we found de-repression of thyroid-specific genes (*NIS*, *Pax*8, *TTF1*, and *TSHR*) in both K18 and SW1736 ShR cells compared with ShN ATCs (Fig. 3f; Supplementary Fig. 2A), suggesting that REGγ might be a critical regulator of the TGF-β signal pathway. Consistently, expression of classical TGF-β target genes, including *PAI-1* and *CTGF*, was markedly higher in SW1736 and K18 ShN than in ShR cells after TGF-β treatments (Supplementary Fig. 2B, C). In contrast, *NIS* and *Pax*8 expression, negatively regulated by REGγ, displayed a marked decrease in a time-dependent manner following the TGF-β activation in ShN cells, while maintained higher levels in ShR cells (Supplementary Fig. 2D, E). The percentage of nuclear Smad3/Smad4 positive cells was significantly decreased with REGγ knockdown (Supplementary Fig. 3A, B). These data indicate that REGγ positively regulates the activity of the TGF-β/Smad signal pathway in ATC cells. Thus, restoration of thyroid-specific gene expression in REGγ-silenced ATC cells may depend on the inhibition of the TGF-β/Smad signal pathway.

### REGγ negatively regulates Smad7 by directly promoting its degradation

**Fig. 4:**
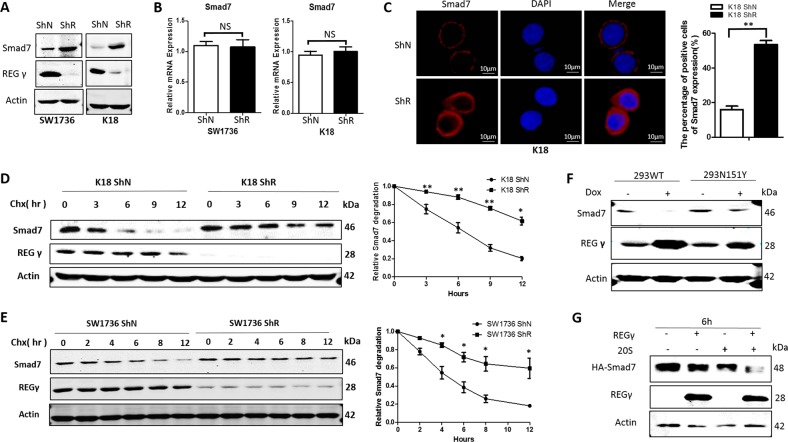
REGγ promotes the degradation of Smad7. **a** REGγ knockdown increased the protein expression of Smad7 by western blot analysis in SW1736 and K18 ShN and ShR ATC cells. **b** The mRNA expression of *Smad7* was similar between ShR and ShN ATC cells by real-time PCR analysis. **c** Immunofluorescence analysis showed stronger staining of Smad7 in K18 ATC cells with knockdown of REGγ. The numbers of Smad7-positive cell number were quantitated. The data were represented as the mean ± SEM. **p < 0.01, ShR vs. ShN. **d**, **e** REGγ regulated Smad7 stability. K18 (**d**) and SW1736 (**e**) ShN and ShR cells were serum starved for 24 h, treated with 5 ng/ml TGF-β for 6 h before the addition of cycloheximide (100 μg/ml) for indicated time and harvested for western blotting analysis. The quantitative results of Smad7 stability were plotted to indicate dynamic changes (two-tailed Student’s *t* test, *n* = 3). *p < 0.05, **p < 0.01, ***p < 0.001, ShR vs. ShN. **f** Induced expression of wild-type (WT), but not inactive mutant N151Y REGγ promotes the degradation of Smad7 in HEK293 cells. REGγ or a mutant form of REGγ was induced in HEK293 cells treated with 1 μg/ml of doxycycline for 48 h and followed by western blot analysis. **g** REGγ directly promoted the degradation of Smad7. In vitro proteolytic analyses were performed using purified REGγ, 20S proteasome, and Smad7 protein at 30 °C for 6 h

Although Smad3 is a key effector regulated by REGγ, it is not a direct target of the REGγ proteasome. By searching for negative regulators of the TGF-β pathway, we observed striking accumulation of Smad7 protein (Fig. 4a), but no increase in mRNA expression (Fig. 4b) in ATC cells with stable RNAi depletion of REGγ. Increased expression of Smad7 in REGγ-deficient K18 and SW1736 cells was validated by immunofluorescence (Fig. 4c; Supplementary Fig. 4A). The percentage of Smad7-positive cells was about threefold higher in both REGγ-ShR K18 and SW1736 cells than in the corresponding ShN cells (Fig. 4c; Supplementary Fig. 4A). These results support the concept that REGγ negatively regulates the protein levels of Smad7 in ATC cells.

To determine if REGγ mediates Smad7 degradation, we analyzed the dynamics of Smad7 stability in the presence of cycloheximide, a protein synthesis inhibitor. In ATC cells lacking REGγ, Smad7 protein is much more stable with a significantly slower decay rate than in the REGγ WT control cells (Fig. 4d, e), suggesting that REGγ is required for degradation of Smad7 in these cells. We carried out gain-of-function experiments using the doxycycline-inducible 293 cells system [10] to overexpress either WT REGγ or the REGγ-N151Y mutant. Induction of REGγ triggered degradation of Smad7 (Fig. 4f), whereas induced expression of the REGγ-N151Y mutant failed to do so (Fig. 4f). We validated physical interactions between REGγ and Smad7 in cells by reciprocal coimmunoprecipitation experiments (Supplementary Fig. 4B, C). To determine if the Smad7 is a direct target of the REGγ proteasome, we performed cell-free proteolysis with purified proteins. Incubation of in vitro-translated Smad7 with 20S proteasome alone or REGγ alone exhibited no significant degradation of Smad7, but a combination of REGγ and 20S proteasome promoted rapid turnover of Smad7 (Fig. 4g). Taken together, our results demonstrate that REGγ can directly interact with Smad7 and promote its degradation in vitro and in SW1736 or K18 ATC cells.

### Clinical relevance of REGγ/Smad7-dependent regulation of thyroid-specific genes in ATC

**Fig. 5:**
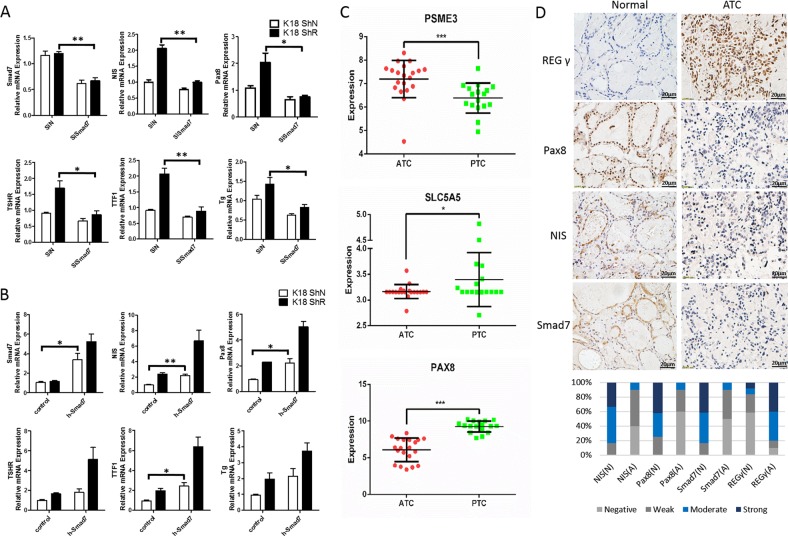
REGγ-Smad7 regulation and correlation with expression of thyroid-specific genes in ATC. **a** Smad7 knockdown blocked the increase of thyroid-specific gene expression induced by REGγ deficiency. K18 ShN and ShR cells were transfected with siRNA against Smad7 for 48 h, and the expression of *NIS*, *Pax8*, *TSHR, Tg*, and *TTF1* were detected by real-time PCR. The data were represented as the mean ± SEM. *N* = 3, *p < 0.05, **p < 0.01, siSmad7 vs. control. **b** Smad7 overexpression promoted the expression of thyroid-specific genes in REGγ ShN K18 ATC cells to levels equivalent to REGγ ShR cells. Cells were transfected with plvx and plvx-GFP-Smad7 vector for 48 h, and the expression of *NIS*, *Pax8*, *TSHR, Tg*, and *TTF1* were detected by real-time PCR. Overexpression efficiency of Smad7 was confirmed by real-time PCR. The data were represented as the mean ± SEM. *N* = 3, *p < 0.05, Smad7 vs. Vector. **c** The expression of REGγ, Pax8, and NIS was analyzed using the microarray data (GSE76039) containing 20 ATC and 17 PTC samples. The data were represented as the mean ± SEM. *p < 0.05, ***p < 0.001. **d** The expression of REGγ, Smad7, Pax8, and NIS as well as the quantitative results of expression profiles in ten human ATC tissues and adjacent normal tissues by immunohistochemistry. Representative images of immunohistochemical staining are shown. Scale bar: 20 μm. Original magnification ×40

To substantiate that REGγ-mediated regulation of thyroid-specific genes is Smad7 dependent, we performed loss- and gain-of-function experiments in ATC cells. Depletion of Smad7 by RNAi markedly downregulated the expression of *NIS*, *Pax8*, *TTF1*, *Tg*, and *TSHR*, which is more dramatic in REGγ-defective ShR than in ShN K18 and SW1736 cells (Fig. 5a; Supplementary Fig. 4D). Depletion of Smad7 eliminated the differences in thyroid-specific gene expression between ShR and ShN ATC cells, suggesting that REGγ action relies on Smad7. In contrast, stable overexpression of Smad7 upregulated the expression of *NIS*, *Pax8*, *TTF1*, *Tg*, and *TSHR*, with a greater increase in REGγ ShN cells than in REGγ ShR K18 or SW1736 ATC cells (Fig. 5b; Supplementary Fig. 4E). Interestingly, overexpression of Smad7 recovered thyroid-specific gene transcription in ShN cells to levels equivalent to those in control ShR cells (Fig. 5b; Supplementary Fig. 4E). These results demonstrate that Smad7 mediates REGγ-dependent regulation of thyroid-specific gene expression in ATC cells.

To understand the clinical significance of the REGγ pathway in ATC, we performed bioinformatics analyses using the NCBI Gene Expression Omnibus database to compare transcription levels of *REGγ, NIS, Pax8*, and *Smad7* in human ATC and PTC samples. Indeed, transcription levels of *REGγ* are higher (*P* < 0.001) in ATC than that in PTC, while NIS and Pax8 were lower in ATC than PTC (Fig. 5c), representing an inverse correlation between REGγ and NIS/Pax8 in human ATC samples. However, *Smad7* mRNA levels showed no significant difference between ATC and PTC (Supplementary Fig. 5A), suggesting a posttranscriptional regulation. Next, we evaluated the protein levels of REGγ, NIS, Pax8, and Smad7 in the human ATC samples by immunohistochemistry comparison with normal thyroid tissues. There was very weak staining of REGγ accompanied with dramatically higher NIS, Pax8, and Smad7 expression in normal human thyroid tissues (Fig. 5d). On the contrary, the REGγ in ATC was significantly elevated, associated with drastically reduced expression of NIS, Pax8, and Smad7 (Fig. 5d). Quantified analysis of the IHC data supports that REGγ expression is negatively correlated with NIS, Pax8, and Smad7 protein levels in human ATC (Fig. 5d), implicating REGγ-Smad7 regulation in ATC tissues.

### REGγ abrogation accentuates ^131^I therapeutic sensitivity to xenograft ATC tumors

**Fig. 6:**
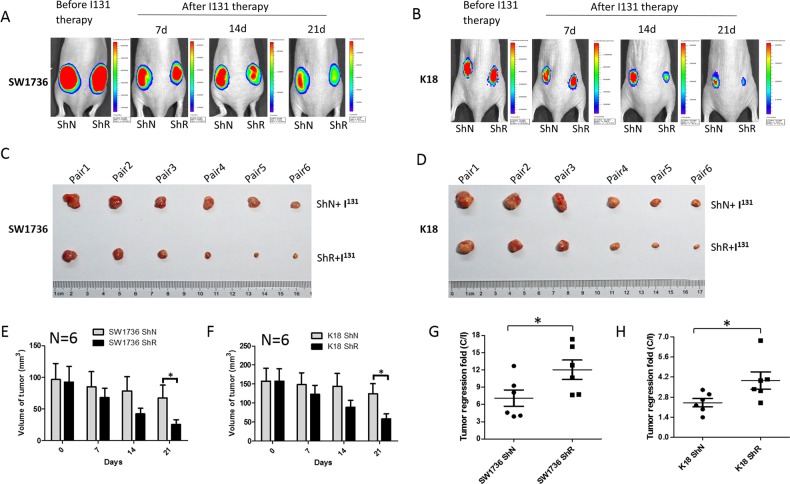
REGγ abrogation sensitizes xenograft ATC tumors to ^131^I therapy. **a**, **b** REGγ knockdown (ShR) in SW1736 (**a**) and K18 (**b**) cells markedly improved the effects of radioiodine therapy compared with the controls (ShN). Each animal with the same size of xenographt tumors from ShN and ShR cells was injected i.p. with 1.5 mCi of carrier-free Na^131^I in 0.1 -ml normal saline. Representative luciferase/luciferin bioluminescence images of tumor-xenografted mice were acquired at the indicated days after the injection of radioactive iodine. Rainbow scales were expressed in radiance (p/s/cm^2^/sr). **c**, **d** Representative photos of xenographt tumors in nude mice by injecting SW1736 (**c**) or K18 (**d**) cells at day 21 after radioiodine therapy. **e**, **f** The tumor volume of NaI^131^ therapy for 0, 7, 14, or 21 days in nude mice was assessed by caliper measurement. The data represented mean ± SEM. *N* = 6, *p < 0.05, ShN vs. ShR. **g**, **h** Tumor regression fold was calculated after radioiodine therapy. REGγ^−/−^ xenografts showed quicker decrease than REGγ^+/+^ ones. The data represented mean ± SEM. *N* = 6, *p < 0.05, **p < 0.01, ShN vs. ShR

Given REGγ-dependent regulation of *NIS* and radioiodine uptake, we evaluated if the therapeutic effect of ^131^I can be modified in an ATC cell xenograft model with altered REGγ expression. Since REGγ-KO tumors grow slower [18, 20], a cohort of ShN and ShR ATC cells were inoculated individually at different time point so that both tumor types would reach 6–8 mm in diameter simultaneously. Following transplantation of paired ShN and ShR ATC tumors to animals, Cherenkov imaging analysis with Na^131^I ensured absorption of radioiodine (Fig. 1d, e). Dynamic evaluation of tumor regression by luciferase activities showed greater decrease in SW1736 ShR than in ShN tumors at days 14 and 21 post therapy (Fig. 6a). Similar results were observed for K18 tumors with a marked reduction of bioluminescence signals in the ShR tumors compared with ShN tumors at days 14 and 21 (Fig. 6b). At the final day of ^131^I treatments, we harvested and measured actual sizes of the paired tumors. Noticeably, immunostaining showed that Pax8, NIS, and Smad7 were still significantly higher in REGγ-deleted tumors than ShN tumors (Supplementary Figs 5B, C). The volume of tumors derived from SW1736 and K18 ShR cells had greater reduction than the tumors from ShN control cells with an approximately threefold difference in tumor volumes on average (Fig. 6c–f; Supplementary Fig. 6A, B). Since the tumor promoting effect of REGγ-positive ShN cells yielded 1.5-fold higher growth rate benefit over ShR cells, we subtracted the growth differences between ^131^I-treated vs. untreated groups to evaluate the net effect of radiotherapy. The results disclosed a greater growth inhibition in treated ShR tumors, indicating more efficient radioiodine therapy in sensitized ShR groups (Fig. 6g, h). Taken together, REGγ inhibition could be a promising direction for treating ATC in the future.

## Discussion

In this study, we have demonstrated that REGγ is an important factor in the regulation of thyroid-specific genes in ATC by directly targeting Smad7 for proteasome-mediated degradation and enhancing the TGF-β signal pathway, which negatively regulates thyroid-specific genes. The significance of REGγ-depletion restoration of ATC differentiation is highlighted by increased uptake of radioiodine and improved effects of ^131^I treatment in the ATC xenograft tumor model. Thus, REGγ appears to be important for driving transformation of ATC from pre-existing differentiated thyroid cancer. This study provides molecular basis that targeting a noncanonical proteasome degradation pathway or directly targeting p-Smad3 may be new directions for ATC therapy.

ATC has the most aggressive progression among thyroid malignancies due to its dedifferentiation; however, the molecular mechanisms of ATC are far from understood. Mutations of various pathways, including p53, Braf, Ras, PTEN/PI3K, and Wnt-β-catenin have been described as potential drivers for ATC [23]. Current therapeutic approaches commonly aim at inhibiting cell proliferation pathways or restoring the function of tumor suppressors. However, single-modality therapy such as Braf inhibitor, PI3K inhibitor, or multimodal treatments has limited effects on ATC, and the mean survival time from diagnosis to death continues to remain at about 6 months [23]. Therefore, there is an urgent need for novel therapies against ATC. Here, we demonstrate for the first time that REGγ is a regulator for *Pax8* and *NIS* transcription. We have provided proof-of-principle evidence that inhibiting REGγ activity greatly improves iodine uptake in ATC cells in vitro and in vivo, which benefits ^131^I therapy in ATC tumor model. Given that REGγ-mediated Smad7 degradation induces NIS repression, combinational therapy to target REGγ and other proteins such as BRAF ^V600E^ might be promising in the future.

It is well established that the TGF-β/Smad pathway plays a critical role in ATC progression and metastasis. Indeed, blocking TGF-β1 inhibits growth and invasion of ATC cells [7, 24]. ATC malignancy caused by TGF-β is probably based on epithelial-to-mesenchymal transition [25, 26]. However, TGF-β also acts as an inhibitor of cell proliferation by suppressing function of c-Myc and enhancing expression of CDK inhibitors such as p15^INK4B^, p21^CIP1^, and p27^KIP1^ [27], particularly exhibiting tumor suppressive effects in healthy noncancerous cells and in early-stage cancerous cells [28]. Since ATC frequently derives from PTC and can co-exist with PTC, in which the TGF-β pathway may function differently than it does in ATC alone. In addition, numerous reports suggest that Smad4 deficiency drives cancer metastasis and tumor malignancy [29–31]. Thus, the function of the TGF-β signal pathway is further complicated by Smad-dependent and Smad-independent pathways [32]. Since TGF-β exerts both tumor suppressive and a metastasis-promoting functions, use of TGF-β receptor inhibitors for ATC patients seems not to be an optimal choice in anticancer therapy.

Smad7 is a key negative regulator of TGF-β signaling by binding the MH2 domain and blocking R-Smad activation [33]. The key function of Smad7 is to control activated Smad2/3 and prevent nuclear translocation. In this study, we observed that the upregulation of *NIS* and *Pax8* after siSmad3 was almost similar to the extent induced by REGγ knockdown in ATC cells, implying that selective targeting Smad3 may be applicable for restoration of the sensitivity to radioiodine therapy. Considering REGγ knockout mice can survive and breed normally, we assume that targeting REGγ may be an alternative option for ATC therapy in the future with less side effects than inhibition of the conventional ubiquitin–proteasome degradation system. In addition, Smad7 has TGF-β-independent function in promoting pluripotency by amplifying STAT3 activation [34]. Thus, our study suggests: (1) targeting Smad7 downstream of TGF-β but not directly affecting receptor levels, may be an economical approach. (2) Proteasome inhibition has proven anticancer effects in ATC [35, 36], the narrow-scoped REGγ proteasome may be specific. It is also noticeable that REGγ was reported to interact with E3-ubiquitin ligase Smurf1 and mediates its degradation [37]. Interestingly, Smad7 also function by binding Smurf2 to TβRI, controlling its turnover and mitigating TGF-β signaling [38]. Given the net effects of Smad7 and Smurf1 on TGF-β/Smad signal pathways are consistent, it is likely that Smad7 and Smurfs may contribute together to the activation of TGF-β signaling affected by REGγ in SW1736 and K18 ATC cells.

Loss of thyroid-specific gene expression, especially *NIS*, contributes to lack of response to radioiodine ablation therapy in ATC, a reason for the high lethality of ATC patients. WTp53 was shown to upregulate NIS expression [39], while Braf^V600E^ and HDAC inhibited its transcription [6, 9]. Coincident with our previous findings [16, 40], abrogation of REGγ enhances p53 levels in multiple cell types. Whether REGγ may also regulate NIS expression in ATC via the p53 pathway is not clear.

**Fig. 7:**
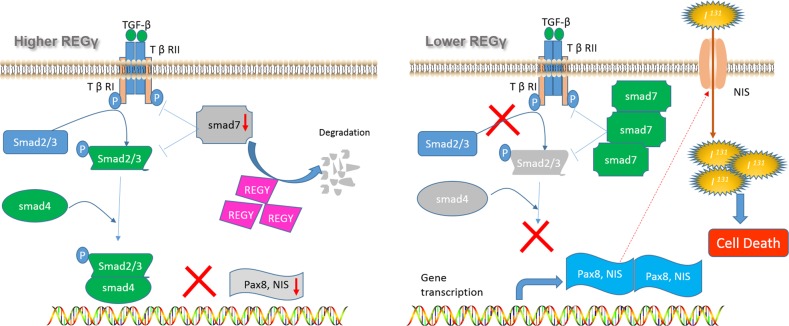
A Model of REGγ-dependent regulation in ATC dedifferentiation. In human ATC cells, overexpression of REGγ promotes the degradation of Smad7, thus activating TGF**-**β signaling and blocking thyroid-specific gene expression. In contrast, cells with REGγ depletion improves Smad7 stability and inhibits Smad2/3 activation, leading to ATC cell re-differentiation and more sensitive responses to radioiodine therapy

Taken together, our results demonstrate a critical role of REGγ in regulating ATC cell differentiation. Antagonizing REGγ greatly restores expression of thyroid-specific genes, intensifies iodide uptake and accentuates cellular sensitivity to radioiodine therapy in ATC (Fig. 7). Molecularly, REGγ acts by activating TGF-β/Smad signaling via proteasome-dependent degradation of Smad7 (Fig. 7), adding an additional layer of the regulatory mechanism in a ubiquitin-independent manner. Therefore, this study presents evidences that blocking REGγ activity may be an alternative therapeutic strategy for anaplastic thyroid carcinoma.

## Supplementary information

Supplemental Figure Legend untracked

Supplemental Figure 1–6
