# Supplementary material for: REGγ ablation impedes dedifferentiation of anaplastic thyroid carcinoma and accentuates radio-therapeutic response by regulating the Smad7-TGF-β pathway
Source: Cell Death Differ. 2019 Jun 26;27(2):497–508. doi: 10.1038/s41418-019-0367-9 (PMC7205985; doi:10.1038/s41418-019-0367-9)
Supplement: Supplementary file 1 — Supplemental Figure Legend untracked [file 41418_2019_367_MOESM1_ESM.docx]

**Supplemental Figure Legends**

**Figure S1. REGγ inhibits thyroid specific gene expression by activating TGF-β/Smad signal pathway. A.** Western blot results for Figure 2C. **B**. The marked nuclear localization of Smad3 in SW1736 cells after treatments of TGF-β was inhibited by REGγ knockdown by immunofluorescence staining. Cells were serum-starved for 24h, then treated with 5ng/ml TGF-β for 6h in the absence or presence 10μM TGF-β receptor inhibitor SB431542. The cells untreated with TGF-β were as vehicle. Nuclei were stained with DAPI. Scale bar, 10 μm. **C.** Quantification of nuclear Smad3 in SW1736 ShN and ShR cells. *p<0.05, ShR versus ShN. **D.** Smad3 silencing in SW1736 ShN ATC cells increased the expression of *NIS*, *Pax8*, *TSHR* and *TTF1* to the similar levels with ShR cells without Smad3 knockdown. Cells were transfected with siRNA against Smad3 for 48h, and the expression of *NIS, Pax8, TSHR* and *TTF1* were detected with Realtime PCR. N = 3, *p<0.05. siSmad3 versus control. **E.** Smad2 and Smad3 overexpression inhibited NIS transcriptional activity. K18 and SW1736 ATC cells were transiently transfected in 12-well plates with 500 ng of NIS–luc, 10 ng of with (+) or without (−) 2.0 μg of Smad2 and Smad3 vector. After 24 hours of transfection, the luciferase activity was measured. Data are represented as the mean ± SEM. *p<0.05, ** p<0.01, Smad2 or Smad3 versus empty vector.

**Figure S2. TGF-β treatments inhibit thyroid-specific gene expression in K18 and SW1736 ATC cells.** **A.** TGF-β treatments inhibit thyroid-specific gene expression in REGγ ShN and ShR ATC cells. Cells were serum-starved for 24h, then treated with or without 5ng/ml TGF-β for 6h. The mRNA expression of *NIS*, *Pax8*, *TSHR* and *TTF1* was detected by Real-time PCR. N = 3, p<0.05. * = TGF-β compare to Control in ShN cells, # = TGF-β compared to Control in ShR cells. **B&C.** K18 (B) and SW1736 (C) ATC cells were sensitive to TGF-β stimulation. Cells were serum-starved for 24h and then treated with 5ng/ml TGF-β for 6h. The mRNA levels of *CTGF* and *PAI-1* at 0, 4, 8 and 12 hours after treatments were measured by Real-time PCR. Data are mean ± SD of triplicate samples. *p<0.05, **p<0.01 TGF-βversus Control. **^#^**p<0.05, ^##^p<0.01, ^###^p<0.001, ShR versus ShN. **D&E.** Real-time PCR analyses were performed to detect the expression of *NIS* and *Pax8* in K18 (D) and SW1736 (E) ATC cells after TGF-β treatments for the indicated time.

**Figure S3**. **REGγ activated TGF-β/Smad signal pathway in K18 and SW1736 ATC cells. A&B.** The nuclear colocalization of Samd3 and Smad4 were repressed by REGγ knockdown in K18 (A) and SW1736 (B) cells. Cells were serum-starved for 24h, then treated with 5ng/ml TGF-β for 6h. Images of double immunofluorescence staining (left panel) and quantification of Smad3 (red) and Smad4 (green) nuclear colocalization (right panel) in ATC cells. Scale bar, 10 μm. *p<0.05, ShR versus ShN.

**Figure S4**. **REGγ can interact with Smad7 and disrupt its stability. A.** Immunofluorescence staining and quantitative analysis for Smad7 in SW1736 ShR and ShN cells**.** *p<0.05, ShR versus ShN. Scale bar, 10 μm. **B.** Smad7 can interacted with REGγ. 293T cells were transfected with 2μg FLAG-Smad7, 2μg FLAG control vector, 2μg HA-REGγ or HA control vector for 72h. Then immunoprecipitations were performed sing anti-FLAG followed by immunoblot using anti–REGγ. **C.** REGγ can interacted with Smad7. 293T cells were transfected with 2μg FLAG-Smad7, 2μg FLAG control vector, 2μg HA-REGγ or HA control vector for 72h. Then immunoprecipitations were performed sing anti-REGγ followed by immunoblot using anti–Flag. **D.** Smad7 knockdown blocked the increase of thyroid-specific gene expression induced by REGγ deficiency. SW1736 cells were transfected with siRNA against Smad7 for 72h, and the expression of *NIS, Pax8, TSHR, Tg* and *TTF1* were detected by Real-time PCR. N = 3, *p<0.05, ** p<0.01. **E.** Smad7 overexpression increased the thyroid-specific gene expression in REGγ ShN cells to the similar levels with REGγ knockdown cells. SW1736 cells were transfected with plvx and plvx-GFP-Smad7 vector for 48h, and the expression of *NIS, Pax8, TSHR, Tg* and *TTF1* were detected by Real-time PCR. N = 3, *p<0.05, ** p<0.01.

**Figure S5.** *REGγ* is negatively with NIS and Pax8. **A**. The mRNA expression of *Smad7* were analyzed in 20 samples of ATC and 17 samples of PTC in accordance with microarray data (GSE76039). **B&C.** Xenograft tumor samples derived from ShR and ShN K18 (B) and SW1736 (C) cells were sectioned and immune-stained with REGγ, Pax8, NIS and Smad7. Representative photomicrographs are shown. Scale bar, 10 μm.

**Figure S6. REGγ knockdown improved the effects of ^131^I therapy.** Each animal with the same size of xenographt tumors was injected i.p. with 1.5 mCi of carrier-free Na^131^I in 0.1 ml normal saline. **A&B** The volume of xenograft tumors derived from SW1736 cells (A) and K18 (B) cells in six pairs of nude mice before and after ^131^I therapy was shown.

**Supplemental Tables**

**Table 1. Sequences of primers used for Q-PCR**

| Gene | Forward(5’→3’) | Reverse(5’→3’) |
| --- | --- | --- |
| *18S (homo)* | GGACACGGACAGGATTGACA | GACATCTAAGGGCATCACAG |
| *NIS (homo)* | GTTCTACACTGACTGCGACCCTC | GCAGCCGAGGTTTGATGAG |
| *Pax8 (homo)* | AAGGTGGTGGAGAAGATTGG | AGGGAGGTTGAATGGTTGC |
| *TTF1 (homo)* | AGCACACGACTCCGTTCTC | GCCCACTTTCTTGTAGCTTTCC |
| *Smad7 (homo)* | GCATTCCTCGGAAGTCAAGAG | CCAGGGGCCAGATAATTCGT |
| *TSHR (homo)* | GGATACTCATAACAACGCTCAT | AGTCATAATGGCTGTCAAA |
| *Smad3 (homo)* | CCATCTCCTACTACGAGCTGAA | CACTGCTGCATTCCTGTTGAC |
| *REGγ (homo)* | AAGGTTGATTCTTTCAGGGAGC | AGTGGATCTGAGTTAGGTCATGG |
| *Tg (homo)* | CTGGCTGAGACAGGTTTGGA | GACTGATTGAACTGCGAGGAA |
| *TPO(homo)* | CTGTCACGCTGGTTATGGC | GCTAGAGACACGAGACTCCTCA |
| *CTGF(homo)* | GACCCAACTATGATGCGAGCC | CCCATCCCACAGGTCTTAGAAC |
| *PAI-1(homo)* | AGTGGACTTTTCAGAGGTGGA | GCCGTTGAAGTAGAGGGCATT |
