# Supplementary material for: REGγ ablation impedes dedifferentiation of anaplastic thyroid carcinoma and accentuates radio-therapeutic response by regulating the Smad7-TGF-β pathway
Source: Cell Death Differ. 2019 Jun 26;27(2):497–508. doi: 10.1038/s41418-019-0367-9 (PMC7205985; doi:10.1038/s41418-019-0367-9)
Supplement: Supplementary file 2 — Supplemental Figure 1–6 [file 41418_2019_367_MOESM2_ESM.pdf]

Supplemental Figures

Supplemental Figure 1

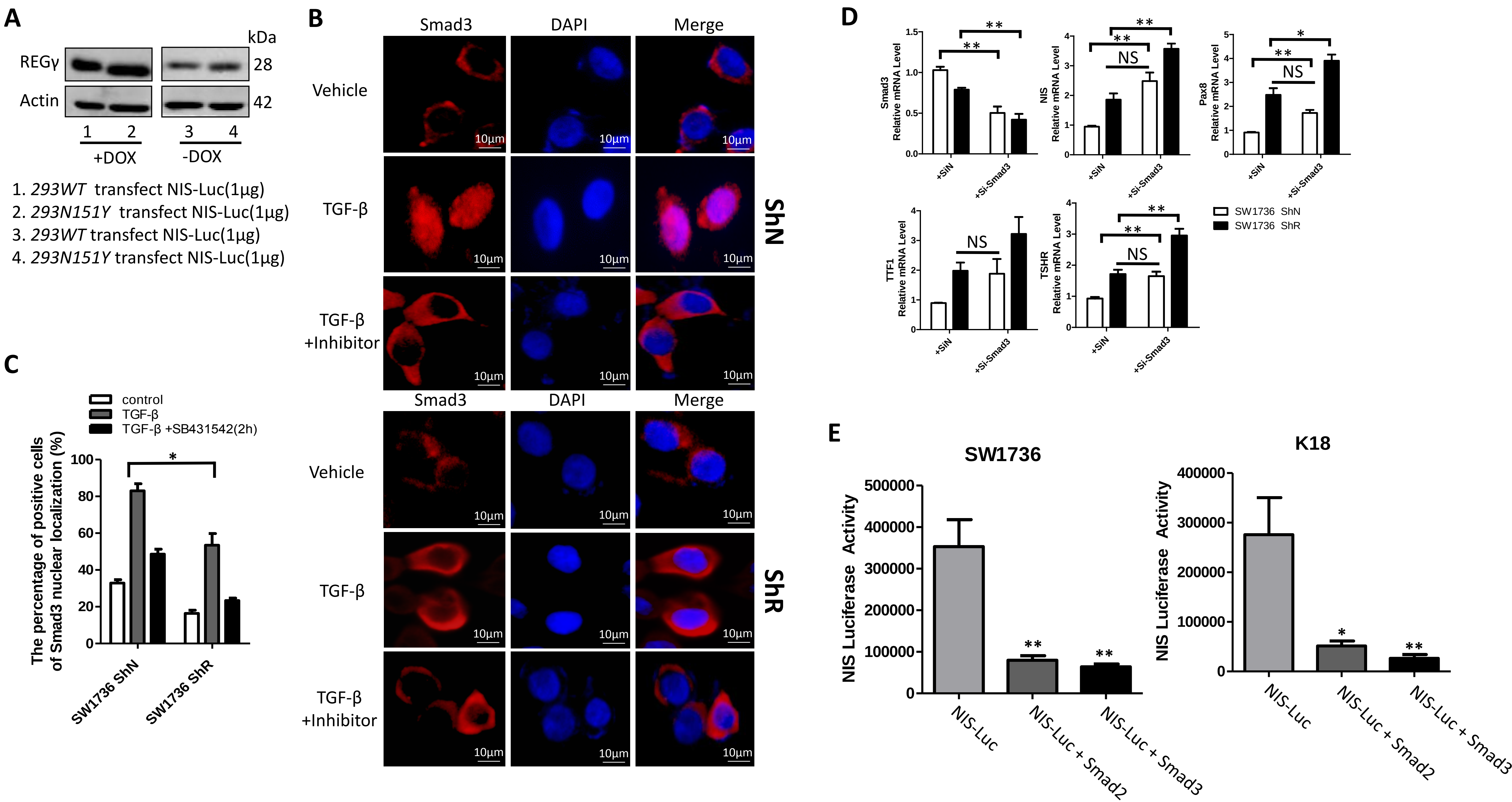

Supplemental Figure 2

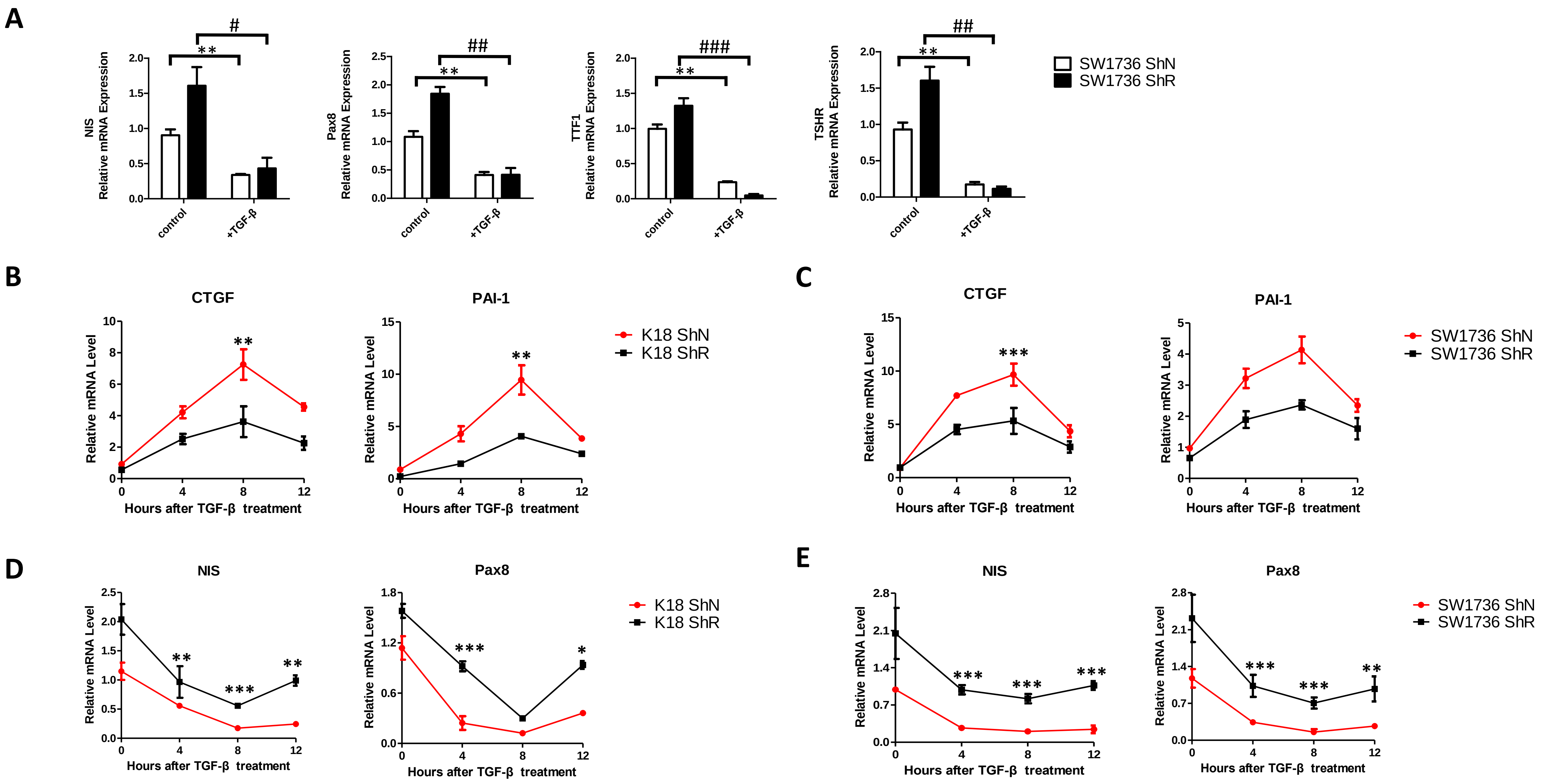

Supplemental Figure 3

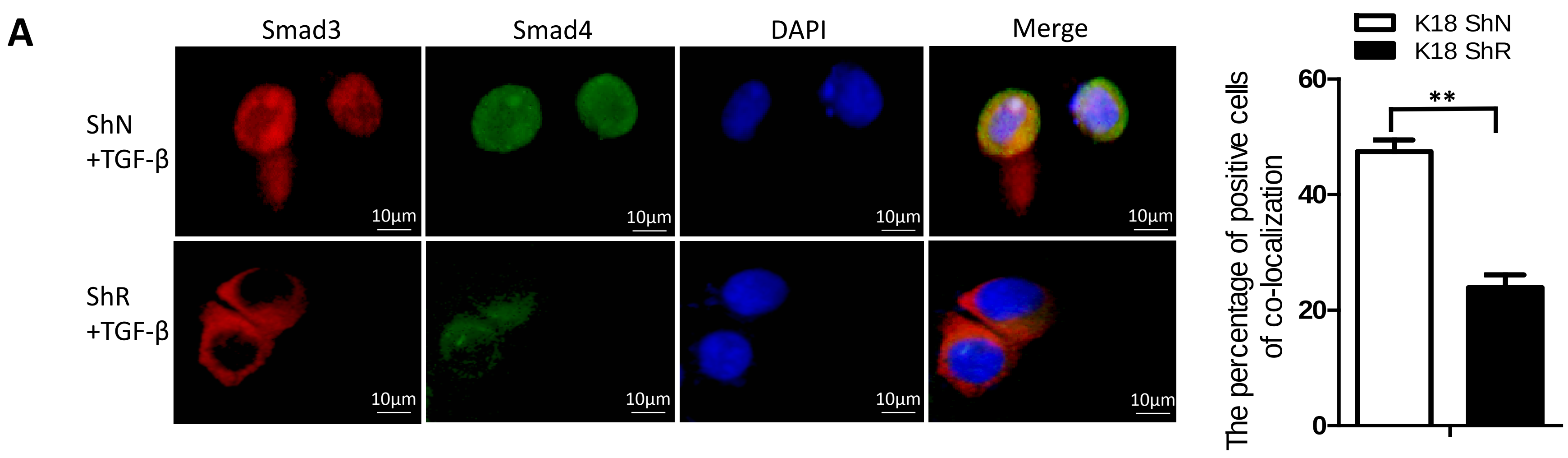

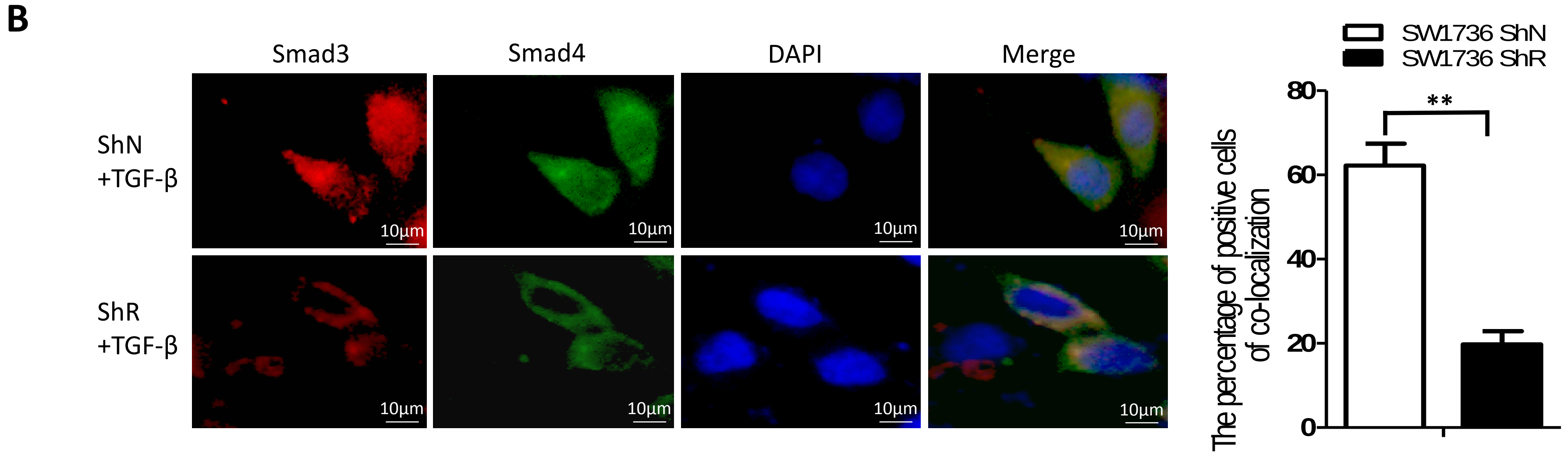

Supplemental Figure 4

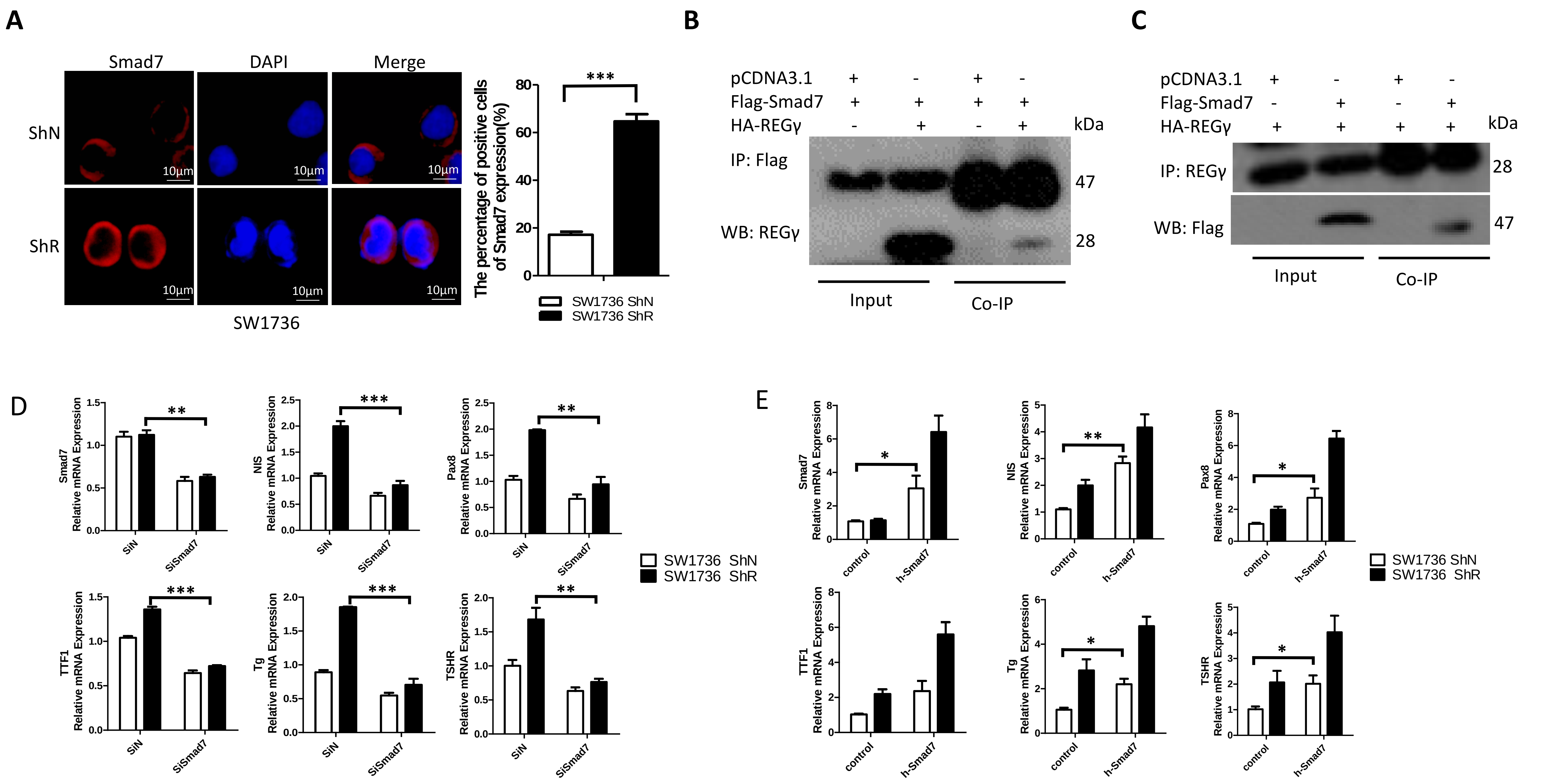

Supplemental Figure 5

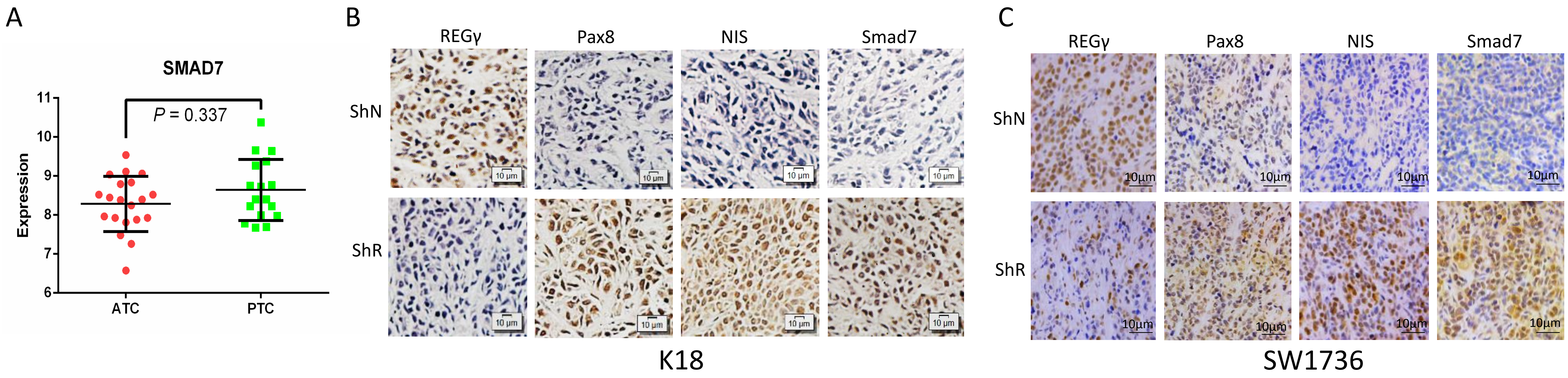

Supplemental Figure 6

A

	SW1736 ShN (mm³)				SW1736 ShR (mm³)			
	0 d	7d	14d	21d	0 d	7d	14d	21d
Pair1	204.5138	195.0388	183.2742	162.0957	197.0197	119.8847	65.88776	57.27245
Pair2	75.91073	72.23407	68.69711	65.91173	74.43137	66.1952	58.34357	36.34634
Pair3	134.8056	101.2104	89.28574	62.24071	123.8924	88.42665	58.11674	22.06966
Pair4	77.56772	62.74029	59.17878	58.5699	85.89929	78.28254	40.03459	19.82323
Pair5	51.24882	45.3591	40.65937	32.04817	42.78669	36.19431	16.58385	9.378958
Pair6	35.63368	34.65634	29.12674	25.0632	32.55619	19.16211	14.63344	9.803976
Average	138.4100	122.8278	113.7524	96.7494	131.7812	91.5022	60.7827	38.5628

B

	K18 ShN (mm³)				K18 ShR (mm³)			
	0 d	7d	14d	21d	0 d	7d	14d	21d
Pair1	179.1775	213.171	243.7029	201.5478	229.0835	186.6143	138.6748	113.4896
Pair2	269.9427	230.5599	216.5075	182.195	224.3279	176.4614	146.6866	72.77405
Pair3	232.177	203.7566	188.2974	158.0379	231.1802	156.959	82.17277	52.42458
Pair4	120.5128	117.9997	103.8773	93.0294	120.8702	98.76895	77.93572	50.89183
Pair5	72.9181	61.72632	56.74469	57.51706	69.50899	51.59571	44.83539	41.47186
Pair6	72.48938	61.72632	56.74469	54.77128	71.656	69.59923	41.96135	22.23566
Average	157.8696	148.8551	144.9777	124.5164	157.7711	123.3331	88.7111	58.88126
